# Understanding repolarization in the intracardiac unipolar electrogram: A long-lasting controversy revisited

**DOI:** 10.3389/fphys.2023.1158003

**Published:** 2023-04-07

**Authors:** Job Stoks, Laura R. Bear, Johan Vijgen, Paul Dendale, Ralf Peeters, Paul G. A. Volders, Matthijs J. M. Cluitmans

**Affiliations:** ^1^ Department of Cardiology, CARIM, Maastricht University Medical Center+, Maastricht, Netherlands; ^2^ Department of Advanced Computing Sciences, Maastricht University, Maastricht, Netherlands; ^3^ Biomedical Research Institute, Hasselt University, Diepenbeek, Belgium; ^4^ IHU Liryc, Electrophysiology and Heart Modeling Institute, Fondation Bordeaux Université, Bordeaux, France

**Keywords:** electrogram, T wave, repolarization, wyatt, alternative method, ECGI, electrophysiology

## Abstract

**Background:** The optimal way to determine repolarization time (RT) from the intracardiac unipolar electrogram (UEG) has been a topic of debate for decades. RT is typically determined by either the Wyatt method or the “alternative method,” which both consider UEG T-wave slope, but differently.

**Objective:** To determine the optimal method to measure RT on the UEG.

**Methods:** Seven pig hearts surrounded by an epicardial sock with 100 electrodes were Langendorff-perfused with selective cannulation of the left anterior descending (LAD) coronary artery and submersed in a torso-shaped tank containing 256 electrodes on the torso surface. Repolarization was prolonged in the non-LAD-regions by infusing dofetilide and shortened in the LAD-region using pinacidil. RT was determined by the Wyatt (t_Wyatt_) and alternative (t_Alt_) methods, in both invasive (recorded with epicardial electrodes) and in non-invasive UEGs (reconstructed with electrocardiographic imaging). t_Wyatt_ and t_Alt_ were compared to local effective refractory period (ERP).

**Results:** With contact mapping, mean absolute error (MAE) of t_Wyatt_ and t_Alt_ vs. ERP were 21 ms and 71 ms, respectively. Positive T-waves typically had an earlier ERP than negative T-waves, in line with theory. t_Wyatt_ -but not t_Alt_-shortened by local infusion of pinacidil. Similar results were found for the non-invasive UEGs (MAE of t_Wyatt_ and t_Alt_ vs. ERP were 30 ms and 92 ms, respectively).

**Conclusion:** The Wyatt method is the most accurate to determine RT from (non) invasive UEGs, based on novel and historical analyses. Using it to determine RT could unify and facilitate repolarization assessment and amplify its role in cardiac electrophysiology.

## 1 Introduction

**FIGURE 1:**
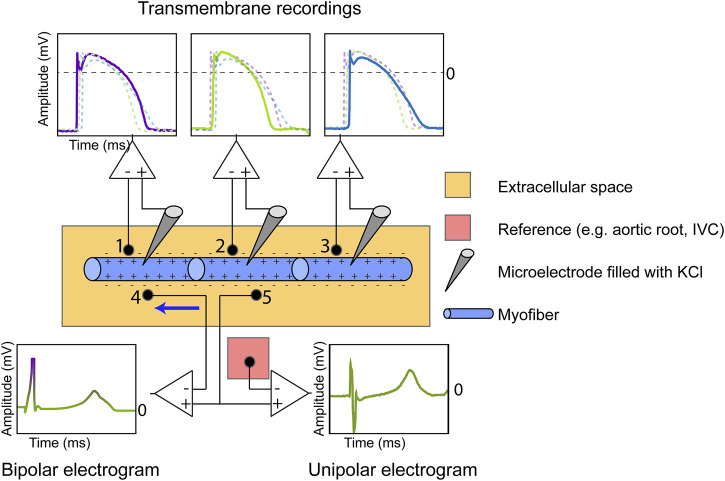
Measurement of the cardiac transmembrane action potential (TMP), bipolar electrogram and unipolar electrogram (UEG). The TMP is recorded by inserting an Ag/AgCl electrode filled with KCl in the intracellular space and using the extracellular potential as a reference. The number of myofibers is reduced for illustration purposes. The extracellular UEG is commonly referenced against electrically inactive tissue (e.g., aortic root, inferior vena cava (IVC)). The bipolar electrogram, measuring current in only one direction (from anode to cathode, see blue arrow), is equal to the subtraction of the UEG measured at location 4 from the UEG measured at location 5.

The intracardiac unipolar electrogram (UEG) is a powerful tool to assess cardiac electrophysiology. It reflects the potential difference between two electrodes in the extracellular space (Haws and Lux, 1990) (Figure 1), and in contrast to its bipolar counterpart, measures electrical activity irrespective of direction. The UEG is routinely employed in the clinical electrophysiology laboratory and basic science. There is good understanding of how electrical activation of cardiac tissue is reflected in the UEG. Conversely, a full appreciation of how repolarization manifests on the UEG remains elusive and has been a topic of debate for decades. This is mainly due to the inherently more complex process of repolarization and inconsistencies in experimental results. Repolarization abnormalities play an important role in arrhythmogenesis, e.g., in long-QT and Brugada syndromes, structural cardiomyopathies and idiopathic ventricular fibrillation (Leong et al., 2018; Cluitmans et al., 2021; Srinivasan et al., 2021). Repolarization heterogeneity can lead to unidirectional conduction block and reentry. (Cluitmans et al., 2021). A unified assessment of repolarization on the UEG could improve our basic and clinical understanding of repolarization in many aspects of electrophysiology, and may increase its role of (non) invasive arrhythmia substrate mapping. Here, we provide novel evidence–and a thorough analysis of previous data–that allows accurate assessment of repolarization from the UEG.

### 1.1 Importance of activation and repolarization assessment

Interest in the UEG focuses on the instants of local electrical activation and recovery, and different methods have been proposed to determine the corresponding activation time (AT) and recovery time (RT) from a UEG. (Durrer and Van der Tweel, 1954; Wyatt et al., 1981; Millar et al., 1985; Steinhaus, 1989; Haws and Lux, 1990; Chen et al., 1991; Gepstein et al., 1997; Punske et al., 2003; Yue et al., 2004; Coronel et al., 2006; Franzone et al., 2007; Potse et al., 2007; Boukens et al., 2017; Wijers et al., 2018). It is widely accepted that the steepest downslope of the QRS-complex of the intracardiac UEG coincides with the moment of local activation, which was first validated in 1954. (Durrer and Van der Tweel, 1954). Assessing AT from the UEG is well-established in arrhythmia studies and is routinely used to determine the origin of premature beats, regions with conduction slowing or low electrical amplitudes, or the exit of a ventricular tachycardia. For a more complete overview of the role of activation mapping in the intracardiac UEG, we refer the reader to (Arora et al., 2010).

The mechanistic role of repolarization in cardiac (patho) physiology is also well understood from experimental studies. Local repolarization heterogeneities (reflected by RT gradients or dispersion) can create a substrate for unidirectional block, a requirement for reentry (Cluitmans et al., 2021). Understanding how to determine RTs from UEGs is crucial to fully comprehend arrhythmia substrates. However, the determination of RT from the UEG is more complex than that of AT, because repolarization is not a propagating wavefront, but a more localized phenomenon that is less dependent on the electrophysiological state of neighbouring myocardium.

### 1.2 Common methods to determine repolarization time

**FIGURE 2:**
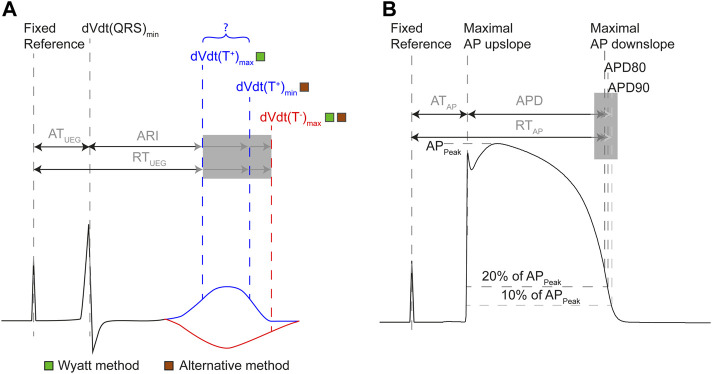
Definitions of terms related to the intracardiac UEG **(A)** and action potential **(B)**. **(A)** AT in the UEG is measured from a fixed reference point (e.g., a common pacing spike) until the steepest downslope of the local QRS complex. RT is measured from the same reference, but its end is defined differently throughout literature. The activation-recovery interval (ARI) is equal to the subtraction of AT from RT. The Wyatt method of determining RT and ARI uses the maximum upslope of the UEG T-wave, regardless of polarity. The alternative method uses the maximum downslope of the T-wave for positive T-waves, and the maximum upslope for negative T-waves. **(B)** In the action potential, AT is defined uniformly: from fixed reference point until the steepest upslope of the action potential (AP). Action potential duration should reflect the same interval as the ARI, but is defined heterogeneously throughout literature (e.g., as the point in time where the cell has repolarized for 80% (APD80), for 90% (APD90), or the TMP reached its maximum downslope). RT in the action potential is measured from the same reference as AT, but its end has been defined differently (analogous to APD definition).

Investigators have predominantly used two distinct methods to determine RT: the Wyatt method and the “alternative” method. The Wyatt method, named after its inventor (Wyatt et al., 1981), defines the end of repolarization as the moment of steepest upslope of the T-wave in the UEG, irrespective of T-wave polarity (Figure 2A). Many investigators have accepted that from a theoretical point of view, the steepest upslope of the UEG T-wave coincides with local RT. (Steinhaus, 1989; Haws and Lux, 1990; Franzone et al., 2007; Potse et al., 2009; Van Duijvenboden et al., 2015; Western et al., 2015). However, some investigators are not convinced that the Wyatt method is optimal for RT determination, and others provide analyses based on both methods due to an apparent lack of consensus. These doubts were fuelled by inconsistencies in early experimental studies (Chen et al., 1991; Gepstein et al., 1997) and a simulation study showing that the Wyatt method could underestimate RTs from positive T-waves under specific conditions such as non-uniform structural properties and a triangular action potential (as during ischemia). (Steinhaus, 1989). This formed the basis for formulating the “alternative” method (Figure 2A), which defines the end of repolarization as the steepest *up*slope for a negative T-wave, but the steepest *down*slope in a positive one. Both methods have been validated experimentally by comparing them to cellular measures such as transmembrane action potential duration at 90% repolarization (APD_90_; Figure 2B), but with varying results and conclusions.

Here, we present novel experimental results comparing the Wyatt and alternative methods directly to the effective refractory period (ERP). ERP is an important measure for cardiac arrhythmogenesis as it directly relates to the potential occurrence of unidirectional block and reentry. We perform this comparison through contact mapping in the setting of repolarization-altering drugs, and we explain these results by addressing theoretical models. Moreover, because non-invasive mapping is increasingly being used (Stoks et al., 2022) and some authors using non-invasive mapping are still in doubt about which method to use, we also investigated how to most accurately determine RT from UEGs from non-invasive electrocardiographic imaging (ECGI). We put our experimental results into historical context, by addressing historical theoretical models and experimental results. Finally, we propose consensus on the optimal approach to determine local RT from the UEG.

## 2 Methods

### 2.1 Experimental protocol and data analysis

**FIGURE 3:**
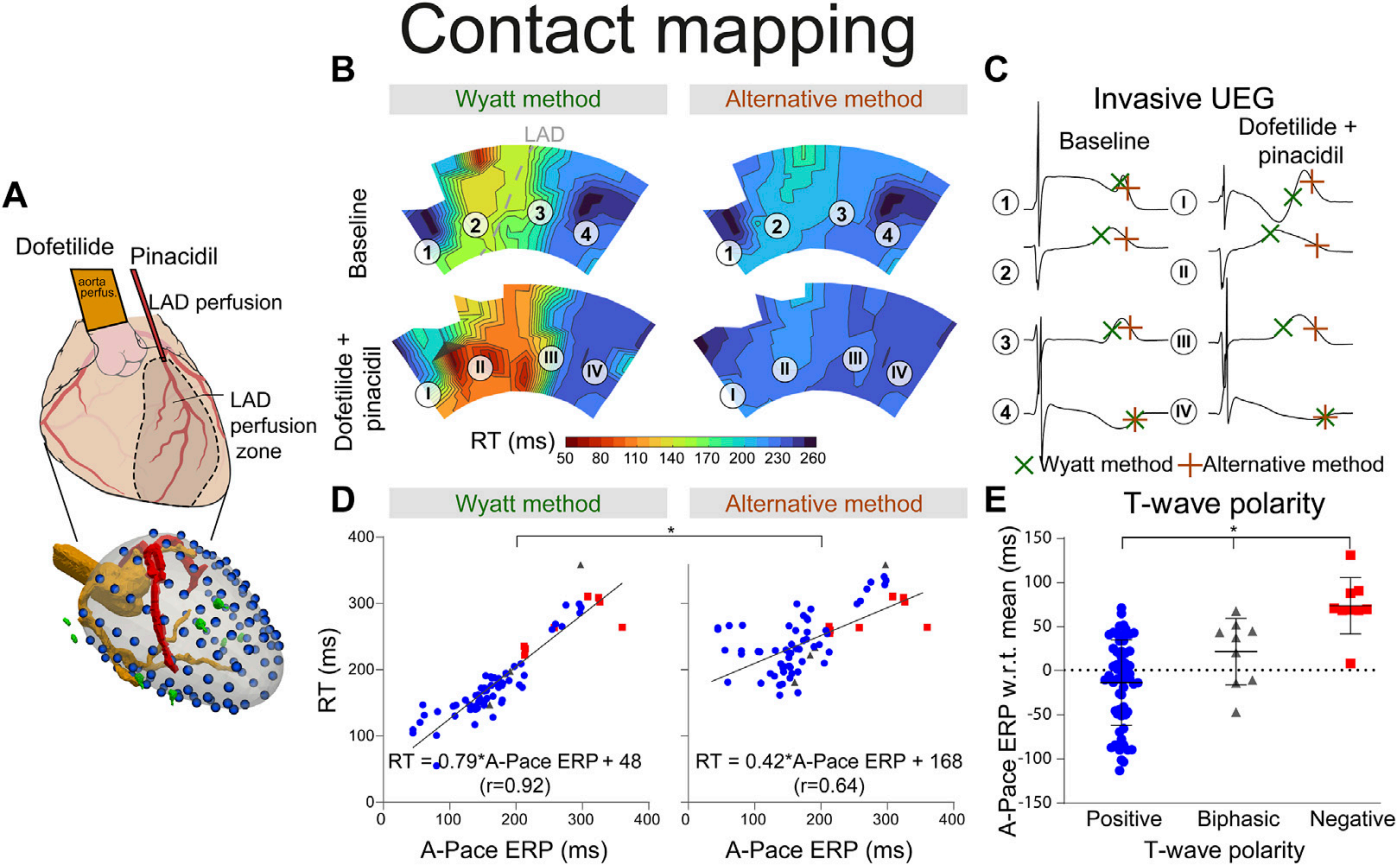
RT determined by the Wyatt and alternative method through contact mapping. **(A)** experimental setup; the LAD was infused with pinacidil which shortens repolarization while the non-LAD region was infused with dofetilide, which prolongs repolarization. Unipolar electrograms were measured with an epicardial sock. **(B)** RT as determined by the Wyatt *versus* alternative method before and during drug infusion. **(C)** electrograms corresponding to different locations in **(B)**. **(D)** linear regression when comparing A-pace ERP (see text) to RT determined by both the Wyatt and alternative method. Positive T-waves are shown in blue, biphasic T-waves in gray, and negative T-waves in red. **(E)** A-pace ERP of positive, biphasic and negative UEG T-waves, with respect to the mean A-pace ERP of the same experiment. Figures **(D,E)** show pooled data of all our experiments with different drug settings.

Procurement was approved by the local ethics committee of Bordeaux CEEA50 and the National Biomedical Agency of France, in accordance with the Directive 2010/63/EU of the European Parliament. Seven male pig hearts were explanted and put on a Langendorff setup with retrograde perfusion of the aorta. A separate cannulation of the left anterior descending (LAD) artery was performed after its ligation, which allowed separate infusion of the LAD-perfused region and the remaining (“aorta-perfused”) myocardium (Figure 3). Hearts were perfused with a 1:9 mixture of blood and Tyrode’s solution, oxygenated with 95%/5% O_2_/CO_2_ (pH 7.4, 37°C). A rigid electrode sock with 100 electrodes (1.8 mm diameter) was placed around the ventricles. The heart was then put in a torso tank as described previously (Bear et al., 2019a), which provided 256 body-surface electrocardiograms, recorded simultaneously with the sock EGMs (both with a 2048 Hz sampling frequency).

A drug-infusion protocol was used to create RT differences using dofetilide at 125 nmol/L and 250 nmol/L, typically in the aorta-perfused region (i.e., everywhere except LAD), and/or pinacidil at 17.5 μmol/L and 35 μmol/L, typically in the LAD. This resulted in regions with pronounced RT prolongation (non-LAD region) and RT shortening (LAD region). Supplementary Table S1 shows which drugs were used for each experiment.

A pair of bipolar pacing electrodes on the atria was used to provide a baseline paced rhythm (“S1 pacing”). After a train of eight atrial S1 beats at 500/600/650 ms, a single ventricular epicardial extrastimulus was provided (“decremental S2 pacing”) at one of three available pairs of bipolar pacing electrodes: the left, right and inferior side of the heart. Near these pacing locations, electrograms were measured prior to measuring the so-called atrial-paced effective refractory period (A-pace ERP). First, the longest interval from atrial S1 to ventricular S2 was determined where the S2 stimulus was not captured. Under that condition, the A-pace ERP was defined as the interval from the body-surface R-peak to the ventricular S2 stimulus, reflecting the moment of local refractoriness at the S2 location with respect to a common, global reference. This was tested with 10-ms decremental intervals. When capture was detected, 1-ms intervals were used to determine the A-pace ERP with higher resolution. AV-conduction was maintained throughout the experiment. Compared to ERP metrics where both S1 and S2 are given on the same (ventricular) location, our A-pace ERP metric captures a more “natural” condition where a ventricular beat may interact with a preceding sinus beat.

Epicardial contact UEGs were filtered by removing 50 Hz powerline noise, and by means of linear detrending. RT was determined by the Wyatt method (t_Wyatt_) and alternative method (t_Alt_) (Figure 2A). Electrograms containing too much noise or ST-segment elevation were disregarded. A-pace ERP was compared to both t_Wyatt_ and t_Alt_ at the electrodes nearest to the pacing electrodes. Metrics were determined relative to a common reference: the R-peak from the body-surface electrocardiogram.

For ECGI, the same experimental protocol was used. Additionally, a coronary angiography with contrast medium was used to obtain the heart geometry, using fluoroscopy. Body-surface electrograms were linearly detrended in combination with a 125 Hz low-pass filter before ECGI was applied. Finally, a potential-based formulation of non-invasive ECGI was used to reconstruct local epicardial UEGs through previously-validated methods. (Cluitmans et al., 2017). t_Wyatt_ and t_Alt_ of non-invasive UEGs were also compared to A-pace ERP.

### 2.2 Statistics

Analyses were performed for both contact UEGs and ECGI. Linear regression was applied with A-pace ERP as the independent variable, and t_Wyatt_ or t_Alt_ as dependent variable. F-tests were used to analyze differences between linear regressions. Kolmogorov-Smirnov testing was used to test for normality. For remaining analyses, when comparing two groups, (non-normally distributed) data were compared using a two-tailed Mann-Whitney-U test. A Kruskall-Wallis test was used for comparing three groups. All tests, with exception of investigating the effect of repolarization-altering drugs vs. baseline, were unpaired and two-tailed. When both drugs were applied simultaneously, the LAD-region and non-LAD region were tested separately from each other. *p* < 0.01 was considered statistically significant.

## 3 Results

**FIGURE 4:**
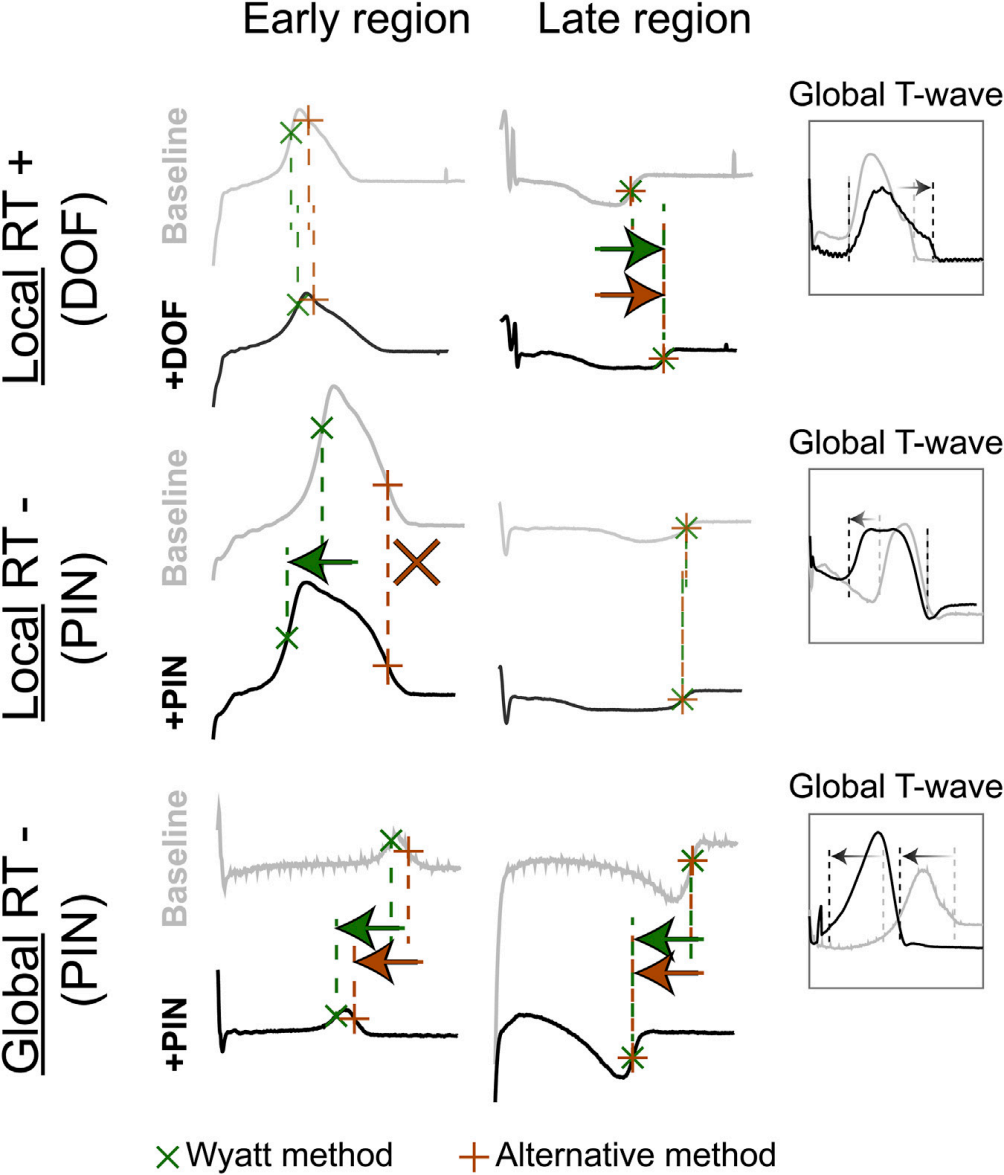
The effect of repolarization-altering drugs on T-wave morphology and RT. Top: repolarization-prolonging dofetilide (DOF) is infused in the late-repolarizing area, causing local RT-prolongation, as captured by both the Wyatt and alternative methods, and the “Global T-wave” (root-mean-square of all epicardial UEGs). Middle: repolarization-shortening pinacidil (PIN) is infused in the early-repolarizing area, causing local RT-shortening, captured by the Wyatt method, but not the alternative method. Bottom: global infusion of pinacidil in the entire heart causes a leftward shift of all T-waves (see global T-wave), which is captured by both methods.

With contact mapping, most electrodes were positioned in the early repolarizing areas: 77% of T-waves were positive, 12% were biphasic and 12% were negative. Examples of RT isochrones as determined by both methods and corresponding electrograms before and after drug infusion are shown in Figures 3B, C. Clearly, t_Wyatt_ -but not t_Alt_-shortened in the region infused with repolarization-shortening pinacidil. Supplementary Tables S2, S3 summarize the effect of drugs on t_Wyatt_ and t_Alt_ over all experiments. t_Wyatt_ and t_Alt_ both prolonged when repolarization-prolonging dofetilide was infused locally, as mostly negative UEG T-waves were affected (Figure 4). When repolarization-shortening pinacidil was infused locally, t_Alt_ often incorrectly prolonged, while t_Wyatt_ shortened, as mostly positive UEG T-waves were affected. When pinacidil was infused throughout the entire heart, both t_Wyatt_ and t_Alt_ shortened, due to a leftward shift of all UEG T-waves.

Mean absolute errors (MAE) of t_Wyatt_ and t_Alt_ relative to A-pace ERP were 21 ms and 71 ms, respectively (*p* < 0.001). For positive UEG T-waves only, MAE was 20 ms for t_Wyatt_ and 78 ms for t_Alt_ (*p* < 0.001). Linear regression using t_Wyatt_ rendered RT_Wyatt_ = 0.79*A-pace ERP+42 (r = 0.92), while linear regression using t_Alt_ rendered RT_Alt_ = 0.42*A-pace ERP+168 (r = 0.64) (*p* < 0.001 comparing linear regressions) (Figure 3D). Positive T-waves typically had an earlier A-pace ERP than negative ones, with biphasic T-waves in between (*p* < 0.001) (Figure 3E). Results of consecutive beats were generally consistent, although the pinpointing of RT from the UEG may be sensitive to slight changes in upslope (for an example, see Supplementary Figure S2).

**FIGURE 5:**
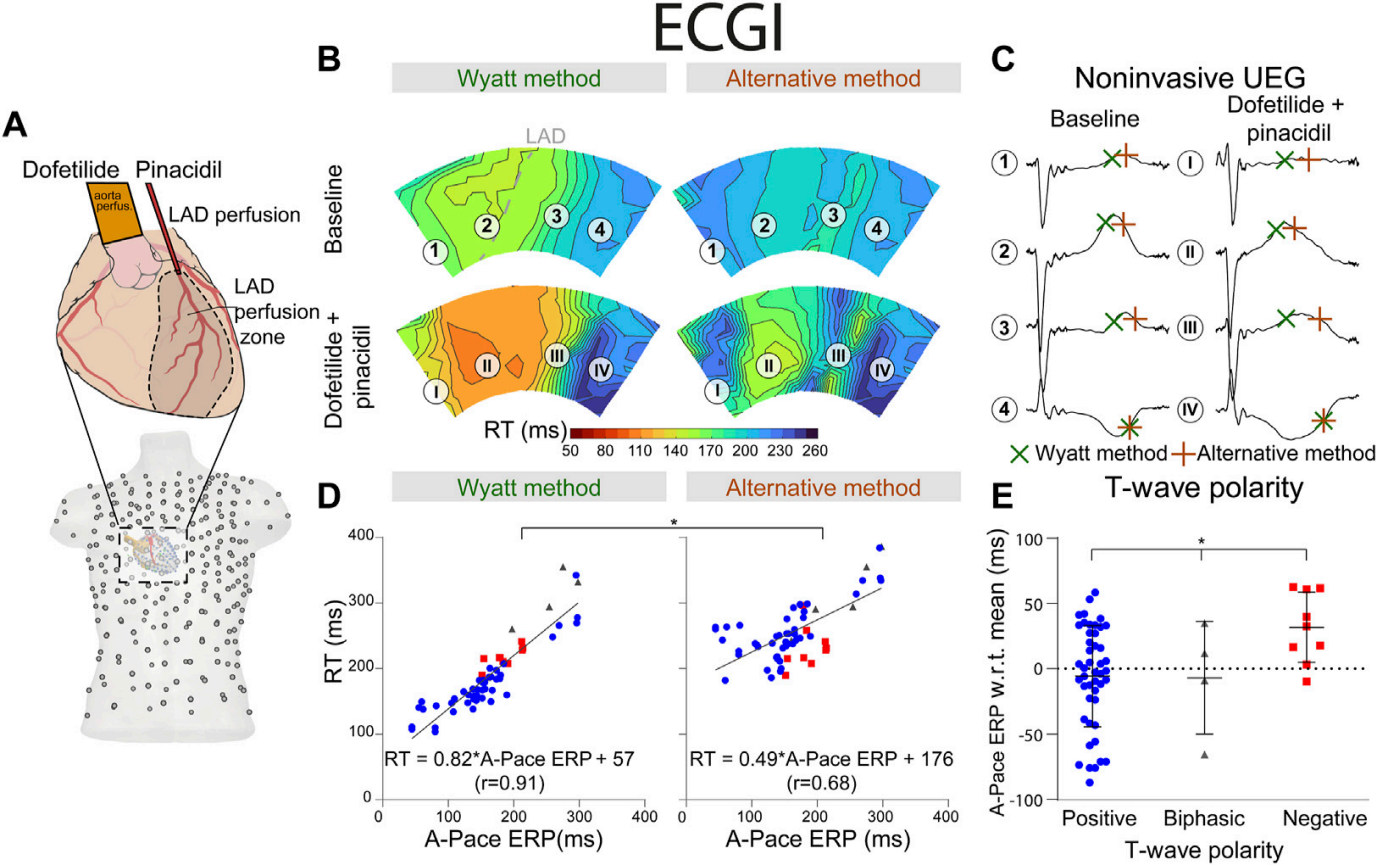
RT determined by the Wyatt and alternative method through non-invasive ECGI. **(A)** experimental setup, similar to the setup in Figure 3. The explanted heart was placed in a torso-shaped tank filled with blood. **(B)** RT as determined by the Wyatt and alternative methods before and after drug infusion. **(C)** electrograms corresponding to different locations in **(B)**. **(D)** linear regression when comparing A-pace ERP (see text) to RT determined by both the Wyatt and alternative method. Positive T-waves are shown in blue, biphasic T-waves in gray, and negative T-waves in red. **(E)** A-pace ERP of positive, biphasic and negative ECGI T-waves, with respect to the mean A-pace ERP of the same experiment. Figures **(D,E)** show pooled data of all our experiments with different drug settings.

Similar results were found for the non-invasive UEGs mapped with ECGI (Figure 5). Reconstructed UEG T-waves (closest to pacing electrodes) were compared to the A-pace ERP. MAE of t_Wyatt_ and t_Alt_ relative to A-pace ERP were 30 ms and 92 ms, respectively (*p* < 0.001). The shortening of t_Wyatt_ caused by pinacidil was much more in line with invasive measurements than the shortening of t_Alt_ (Figures 3, 5). For positive T-waves only, MAE was 32 ms for t_Wyatt_ and 98 ms for t_Alt_ (*p* < 0.001). Linear regression for t_Wyatt_ rendered RT_Wyatt_ = 0.82*A-pace ERP+57 (r = 0.91), linear regression with t_Alt_ rendered RT_Alt_ = 0.49*A-pace ERP+176 (r = 0.68) (*p* < 0.001 comparing linear regressions) (Figure 5D). Compared to the mean measured A-pace ERP in the same experiment, local A-pace ERP was −6 ± 39 ms for positive ECGI T-waves, −7 ± 43 ms for biphasic T-waves and 32 ± 27 ms for negative T-waves (*p* < 0.001) (Figure 5E).

## 4 Discussion

Our novel experimental data show that the correlation between A-pace ERP (which we consider the most relevant ground truth for RT) and the Wyatt method is much higher than between A-pace ERP and the alternative method, and closer to the line of unity. Moreover, as in theoretical models, UEG T-wave polarity relates to RT, with UEG T-waves becoming increasingly negative as RT prolongs. Our new observations also show that local infusion of repolarization-shortening drugs was captured by the Wyatt method, while the alternative method often showed a prolongation of RT. Furthermore, our comparison of local vs. global infusion of repolarization-altering drugs provides additional mechanistic confirmation of theoretical models investigating RT in the UEG. The Wyatt method also performed consistently between consecutive beats.

For the first time, we show that the Wyatt method also reflects RT more accurately than the alternative method through ECGI. With ECGI, the relationship between T-wave polarity and RT was less evident, relating to earlier work showing that ECGI can reliably map RT and related gradients (Cluitmans et al., 2017; Bear et al., 2021), but biphasic UEG T-waves can be challenging to reconstruct and may be rendered flat through ECGI. (Bear et al., 2019b).

### 4.1 Historical experimental and theoretical studies

**FIGURE 6:**
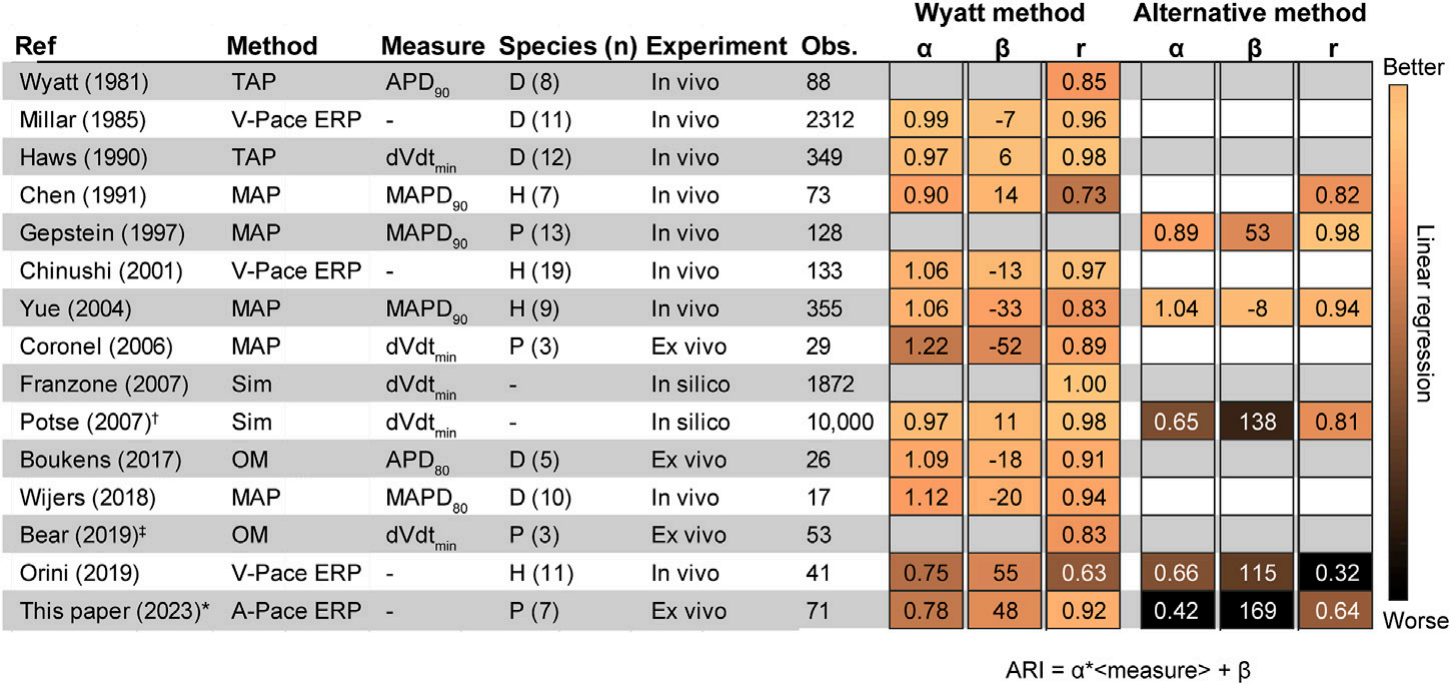
Literature results comparing the invasive unipolar electrogram (UEG) T-wave to different outcome measures. (Wyatt et al., 1981; Millar et al., 1985; Haws and Lux, 1990; Chen et al., 1991; Gepstein et al., 1997; Chinushi et al., 2001; Yue et al., 2004; Coronel et al., 2006; Franzone et al., 2007; Potse et al., 2007; Boukens et al., 2017; Wijers et al., 2018; Bear et al., 2019a; Orini et al., 2019). The Figure shows Pearson’s r, the slope (α) and intercept (β) according to the equation ARI = α ⋅<local measure>+ β, i.e., for Orini (2019): RT = α ⋅V-pace ERP+ β (Figures 3, 4 for an example). †: Analysis only for positive T-waves. ‡: RT instead of ARI (Figure 2A). If pooled data on r, α and/or β were not available, a weighted average was calculated for separate experiments. D: Dog. H: Human. MAP: monophasic action potential. Obs.: number of observations. OM: Optical mapping. P: Pig. Sim: Simulation. TMP: transmembrane action potential. V-pace ERP/A-pace ERP: ventricular/atrial-paced effective refractory period (see text).

Experimental studies validating either the Wyatt or alternative method by determining RT against ground truth-measurements are summarized in Figure 6. Generally, most studies agree that the Wyatt method outperforms the alternative method.

Studies validating the Wyatt method were performed in a variety of conditions: in Langendorff-perfused pig hearts (Coronel et al., 2006; Bear et al., 2019a), left-ventricular canine wedge preparations (Boukens et al., 2017), *in-vivo* dogs (Wyatt et al., 1981; Millar et al., 1985; Haws and Lux, 1990) and humans (Chinushi et al., 2001; Orini et al., 2019). These experiments were done under a variety of conditions to alter repolarization: control, different pacing cycle lengths (Wyatt et al., 1981; Millar et al., 1985; Chinushi et al., 2001; Boukens et al., 2017; Orini et al., 2019), coronary occlusion and reperfusion (Wyatt et al., 1981), epinephrine infusion (Wyatt et al., 1981; Millar et al., 1985), sympathetic nerve stimulation (Millar et al., 1985; Haws and Lux, 1990), local warming (Haws and Lux, 1990; Coronel et al., 2006) and cooling (Coronel et al., 2006), graded myocardial perfusion (Haws and Lux, 1990), during dl-sotalol infusion (Chinushi et al., 2001), in different locations on the heart.

In support of the Wyatt method, localized warming was found to shorten APD together with the UEG T-wave flipping from negative to positive, thus also shortening UEG RT according to that method (Haws and Lux, 1990; Coronel et al., 2006). Localized cooling produced the opposite effect. In *ex-vivo* experiments on isolated pig hearts, Coronel et al. (Coronel et al., 2006) showed that local activity is terminated at the peak of the positive local UEG T-wave, illustrating that RTs defined by the alternative method are by definition later than the true RT for positive T-waves. Orini et al. (Orini et al., 2019) used both the Wyatt and alternative methods to determine correlation between activation-recovery interval (ARI, the subtraction of AT from RT; see Figure 2) and ERP at different sites in 11 patients with structurally normal hearts, the error of ARI *versus* ERP being 10.1 ± 15.5 ms for the Wyatt method, but −56.8 ± 16.2 ms for the alternative method (Orini et al., 2019).

The alternative method has been evaluated in fewer experimental studies than the Wyatt method. Experiments were performed in *in-vivo* pigs, (Gepstein et al., 1997), humans with monomorphic ventricular tachycardia (Yue et al., 2004) and humans with right-ventricular hypertrophy (Chen et al., 1991) during normally-conducted sinus rhythm (Chen et al., 1991) and pacing with variable cycle lengths (Gepstein et al., 1997; Yue et al., 2004).

Two clinical studies (Chen et al., 1991; Yue et al., 2004) yielded results in support of the alternative method over the Wyatt method. In patients with right-ventricular hypertrophy, a better overall correlation between ARI and monophasic APD_90_ (MAPD_90_) was found when using the alternative method, compared to the Wyatt method (0.82 vs. 0.73, respectively). (Chen et al., 1991). In patients with monomorphic ventricular tachycardia and a normal left-ventricular ejection fraction, use of the alternative method (compared to the Wyatt method) resulted in an increase of ARI-MAPD_90_ correlation from 0.83 to 0.94 and decreased MAE. (Yue et al., 2004). However, in that study, non-contact mapping was used, which was shown to correlate poorly with contact electrograms (Schilling et al., 1998) and the method was not validated for intracardiac RT determination. (Coronel et al., 2007).

The theoretical underpinnings of the UEG have been extensively studied by comparing it with transmembrane potentials (TMPs) at the microstructural level. (Steinhaus, 1989; Haws and Lux, 1990). Bidomain equations have been used to translate these one-dimensional models to a three-dimensional heart. Theoretical models (Steinhaus, 1989; Franzone et al., 2007; Potse et al., 2009) agree that the Wyatt method forms a solid theoretical basis for estimating the end of cellular repolarization from the UEG. However, these purely mathematical approaches are not intuitively straightforward to understand and only provide a physical, not physiological, explanation for the Wyatt method.

**FIGURE 7:**
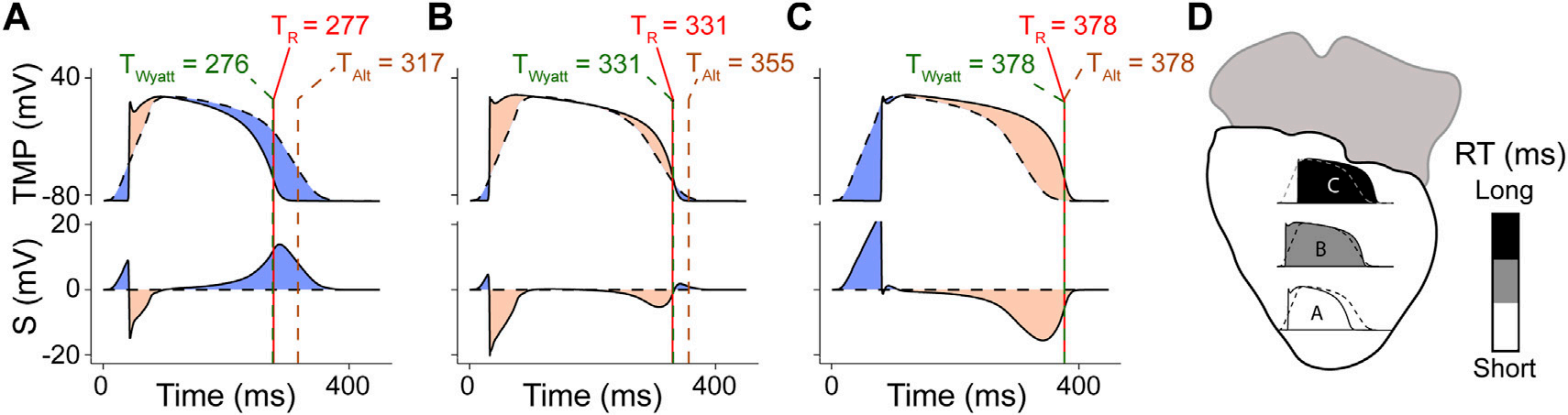
Model for improved interpretation of the UEG, as proposed by Potse et al. (Potse et al., 2009), and slightly adapted from the original version. Panels **(A–C)** denote different locations (highlighted in panel **(D)**). The top frame of each panel shows simulated TMPs from 3 different locations in the model (solid line) and the location-independent mean action potential (dashed). The second frame shows S, i.e., the UEG itself (computed with the model), which is the subtraction of both. Each red vertical line indicates TR, i.e., the instant of minimum dVdt in the TMP. Each of these lines is half obscured by a dashed green line, which indicates tWyatt, leading to a green-red dashed line. Each dashed brown vertical line indicates tAlt. **(A)** Action potential and UEG for a short-RT-region, resulting in a positive UEG T-wave. **(B)** intermediate-RT-region, resulting in a biphasic T-wave. **(C)** long-RT-region, resulting in a negative T-wave.

More recently, Potse et al. (Potse et al., 2007; Potse et al., 2009) developed a simpler model for UEG interpretation which agrees with the Wyatt method, validated with the more complex bidomain equations. (Orini et al., 2018). In this model, the UEG is defined as the difference between the local TMP and the average TMP from the myocardial surface. For *relatively* early-repolarizing myocardium, the local TMP is less negative than the average TMP, leading to a positive T-wave in the UEG. Conversely, for *relatively* late-repolarizing tissue, the local TMP is more negative than the average TMP, leading to a negative T-wave (Figure 7). This model was later validated against *in-vivo* contact mapping. (Orini et al., 2018). Our results are in agreement with these previous observations (Figures 3E, 5E). Moreover, our experiments further confirmed the relationship between UEG T-wave upslope and repolarization pattern: *global* infusion of pinacidil caused a leftward shift of *all* T-waves and their upslopes, thereby maintaining the repolarization pattern (i.e., the relative relationship between early and late RT remained the same). In contrast, *local* infusion of repolarization-altering drugs caused only *local* changes in T-wave upslope, thereby altering the repolarization pattern (Figure 4).

### 4.2 Experimental conventions and challenges

Multiple factors can influence the local unipolar T-wave and may explain the contradicting data from a small subset of the historical experiments.

First, larger electrode size and electrode-to-tissue distance may alter UEG T-wave morphology since both aspects increase the effective field-of-view of the electrode to a larger area, which could affect ARI-APD correlation in favor of the alternative method. (Western et al., 2015). Additionally, 2 Hz high-pass electrogram filtering (often used for activation mapping) has been shown to flip T-wave polarity in some cases, which severely affects RT determination and could cause results to lean in favor of the alternative method. (Langfield et al., 2021). Consequently, high-pass filters should be used with great caution for repolarization mapping (i.e., use a low cut-off frequency). (Langfield et al., 2021).

Secondly, heterogeneous definitions and measurement approaches have been used throughout the literature. For example, different gold standards have been used to compare UEG RT with, such as the local extracellular monophasic action potential (Chen et al., 1991; Gepstein et al., 1997; Yue et al., 2004; Coronel et al., 2006; Wijers et al., 2018), the more accurate TMP(5) (which can hardly be obtained in a beating heart) and optical mapping together with mechanical uncouplers. (Boukens et al., 2017; Bear et al., 2019a). Some authors compared ARI to ERP, of which the latter is a robust measure in terms of arrhythmogenesis as it directly links to conduction block.

Additionally, even in ground-truth measurements, RT is not uniformly defined: e.g., as the maximum downslope of the action potential (Steinhaus, 1989; Haws and Lux, 1990; Coronel et al., 2006; Potse et al., 2009), (M) APD_90_ (Wyatt et al., 1981; Gepstein et al., 1997; Yue et al., 2004), (M) APD_80_ (Boukens et al., 2017; Wijers et al., 2018) and the end of the action potential (Yue et al., 2004) (Figure 2B). Moreover, species differences (dog/pig/human), recording site (endocardial/epicardial) and experimental model (*in vivo*/*ex vivo*/*in silico*) may play a role. However, the general conclusion of the Wyatt method outperforming the alternative method in historical literature still holds strong when isolating these factors (Supplementary Figure S1).

Moreover, the RT is typically measured from a global reference, e.g., a pacing spike, until the end of local repolarization (Figure 2A). However, most studies have compared APD to ARI, i.e., the subtraction of AT from RT. AT from the UEG and TMP can correlate poorly in certain conditions. (Cluitmans et al., 2022). Consequently, a comparison between APD and ARI may include measurement error in AT and could render different results than a comparison between RT (from UEG) and RT (from TMP).

Besides the Wyatt method and the alternative method to determine local RT from the UEG, other signal-analysis methods have been suggested, such as T-wave area-based methods (Potse et al., 2009) or spatiotemporal methods (Cluitmans et al., 2022). These methods seem promising to determine RT in cases where slope-based methods are challenging, for example, when noise levels are high or UEG T-wave amplitude is low. However, these methods have not been studied nearly as extensively as either slope-based method.

### 4.3 Study limitations

We selected the A-pace ERP as a gold standard for RT. This does differ from most of the historical literature where other gold standards were used. However, we believe ERP is the most robust measure for RT in terms of arrhythmogenesis, since it defines the ability of tissue to block conduction. Secondly, we did not investigate scenarios of postrepolarization refractoriness (PRR) which may affect the relationship between ERP and RT. However, as PRR occurs *beyond* termination of local repolarization (Coronel et al., 2012), we do not expect it to be reflected in the UEG T-wave. Thirdly, the study was performed in pigs, not in humans. However, we believe that the physical and physiological mechanisms behind RT-determination do not differ between species, as also supported Supplementary Figure S1. Furthermore, ERP was determined for a limited number of places in the heart because of the exhaustive experimental protocol. Lastly, we chose to investigate primarily the tissue with positive T-waves, because it causes a different RT by using either method. As such, this helped emphasize the differences between both methods in our analyses.

### 4.4 Application to basic and clinical arrhythmogenesis

In support of our novel experimental results, the collective results from previous experimental, clinical and computational studies show that the Wyatt method outperforms the alternative method in determining RT from the UEG (Figures 3, 4, 6, and 7), which is also supported by a theoretical understanding of the UEG T-wave (Figure 7): positive T-waves are found in early-repolarizing tissue, while negative T-waves are found in late-repolarizing tissue. This aspect has been found widespread experimentally (Millar et al., 1985; Coronel et al., 2006; Van Duijvenboden et al., 2015; Orini et al., 2018) even by authors who claim the alternative method should be used (Gepstein et al., 1997), except for Chen et al. (Chen et al., 1991)

Our novel findings and our historical overview support a unified repolarization assessment, thereby enhancing our understanding of mechanisms of repolarization. This augments our knowledge of repolarization in both structural and functional arrhythmias, since many arrhythmias are caused by local heterogeneities of repolarization–which can lead to unidirectional conduction block and reentry. A unified repolarization assessment may increase the role of invasive and non-invasive repolarization mapping.

## 5 Conclusion

We scrutinized our novel experimental results and historical experimental and theoretical studies to resolve the controversy between the Wyatt and alternative methods for determining RT from UEGs. The Wyatt method outperforms the alternative method not only on a theoretical basis but also in our and historical experimental data. Our results support that the Wyatt method provides a strong basis for RT determination from the invasive UEG and non-invasive (ECGI) UEG. Using it to determine RT could unify and facilitate repolarization assessment and amplify its role in basic and clinical electrophysiology.

## Data Availability

Upon reasonable request, the raw data supporting the conclusion of this article will be made available by the authors, without undue reservation.
