# Supplementary material for: Understanding repolarization in the intracardiac unipolar electrogram: A long-lasting controversy revisited
Source: Front Physiol. 2023 Apr 7;14:1158003. doi: 10.3389/fphys.2023.1158003 (PMC10119409; doi:10.3389/fphys.2023.1158003)
Supplement: Supplementary file 1 [file DataSheet1.DOCX]

Supplementary Material

Understanding repolarization in the intracardiac unipolar electrogram: a long-lasting controversy revisited

**Job Stoks, MSc, Laura Bear, PhD, Johan Vijgen, MD, Paul Dendale, MD PhD, Ralf Peeters, PhD, Paul GA Volders, MD PhD, Matthijs JM Cluitmans, MD PhD***

**Correspondence:** Matthijs JM Cluitmans: [m.cluitmans@maastrichtuniversity.nl](mailto:m.cluitmans@maastrichtuniversity.nl)

# Supplementary Tables

| **Supplementary Table 1.** Drugs used in each experiment. Exp: experiment; DOF; dofetilide; PIN; pinacidil. “Yes” (in green) indicates that a drug setting was used, “No” (in red) indicates that a setting was not used. Dofetilide was typically infused in the aorta-perfused region (i.e., everywhere except LAD), pinacidil typically only in the LAD, with exception of experiment #7. ^†:^ global infusion of pinacidil, not only in LAD-region. |
| --- |
| \| **Exp. ID** \| **Baseline** \| **Isolated DOF** \| **Isolated PIN** \| **DOF and PIN** \| \| --- \| --- \| --- \| --- \| --- \| \| 1 \| Yes \| Yes \| No \| Yes \| \| 2 \| Yes \| Yes \| No \| No \| \| 3 \| Yes \| No \| Yes \| No \| \| 4 \| Yes \| Yes \| No \| Yes \| \| 5 \| Yes \| Yes \| No \| No \| \| 6 \| Yes \| Yes \| No \| Yes \| \| 7^†^ \| Yes \| No \| Yes \| No \| |

| **Supplementary Table 2.** Differences between RT (delta-RT, in milliseconds) according to the Wyatt method after repolarization-altering infusion of drugs (repolarization-shortening pinacidil (PIN) and repolarization-prolonging dofetilide (DOF)), with respect to baseline situation without drugs. Values are noted as median [first quartile – third quartile]. A positive value indicates a prolongation of RT by the effect of drugs, with respect to baseline. Asterisks (*) denote significant differences (P<0.01, one-tailed test). Colors denote whether the effect of the drug was correctly identified by delta-RT (green for yes, red for no). For experiment #7 (^†^), pinacidil was infused globally. |
| --- |
| \| **Delta-RT (ms)** \| **Isolated DOF** \| **Isolated PIN** \| **DOF and PIN** \| \| \| --- \| --- \| --- \| --- \| --- \| \| **Exp. ID** \| **Non-LAD region** \| **LAD region** \| **Non-LAD region (DOF)** \| **LAD region (PIN)** \| \| 1 \| 37 [31 – 49]* \| N/A \| 10 [7 – 16]* \| -37 [-56 – -32]* \| \| 2 \| 51 [46 – 56]* \| N/A \| N/A \| N/A \| \| 3 \| N/A \| -52 [-63 – -45]* \| N/A \| N/A \| \| 4 \| 40 [36 – 44]* \| N/A \| 26 [22 – 31]* \| -77 [-88 – -71]* \| \| 5 \| 49 [33 – 52]* \| N/A \| N/A \| N/A \| \| 6 \| 40 [27 – 53]* \| N/A \| 14 [-8 – 26]* \| -10 [-20 – -7] \| \| 7^†^ \| N/A \| -88 [-99 – -77]* \| N/A \| N/A \| |

| **Supplementary Table 3.** Differences between RT (delta-RT, in milliseconds) according to the alternative method after repolarization-altering infusion of drugs, with respect to baseline situation without drugs. Values are noted as median [first quartile – third quartile]. A positive value indicates a prolongation of RT by the effect of drugs, with respect to baseline. Asterisks (*) denote significant differences (P<0.01, one-tailed test). Colors denote whether the effect of the drug was correctly identified by delta-RT (green for yes, red for no). For experiment #7 (^†^), pinacidil was infused globally. |
| --- |
| \| **Delta-RT (ms)** \| **Isolated DOF** \| **Isolated PIN** \| **DOF and PIN** \| \| \| --- \| --- \| --- \| --- \| --- \| \| **Exp. ID** \| **Non-LAD region** \| **LAD region** \| **Non-LAD region (DOF)** \| **LAD region (PIN)** \| \| 1 \| 38 [33 – 49]* \| N/A \| 15 [10 – 17]* \| 16 [14 – 22] \| \| 2 \| 54 [49 – 62]* \| N/A \| N/A \| N/A \| \| 3 \| N/A \| -8 [-10 – -3]* \| N/A \| N/A \| \| 4 \| 40 [38 – 44]* \| N/A \| 26 [24 – 29]* \| 22 [14 – 30] \| \| 5 \| 51 [45 – 61]* \| N/A \| N/A \| N/A \| \| 6 \| 35 [22 – 40]* \| N/A \| 37 [26 – 42]* \| 21 [13 – 32] \| \| 7 \| N/A \| -93 [-113 – 82]* \| N/A \| N/A \| |

# Supplementary Figures

| 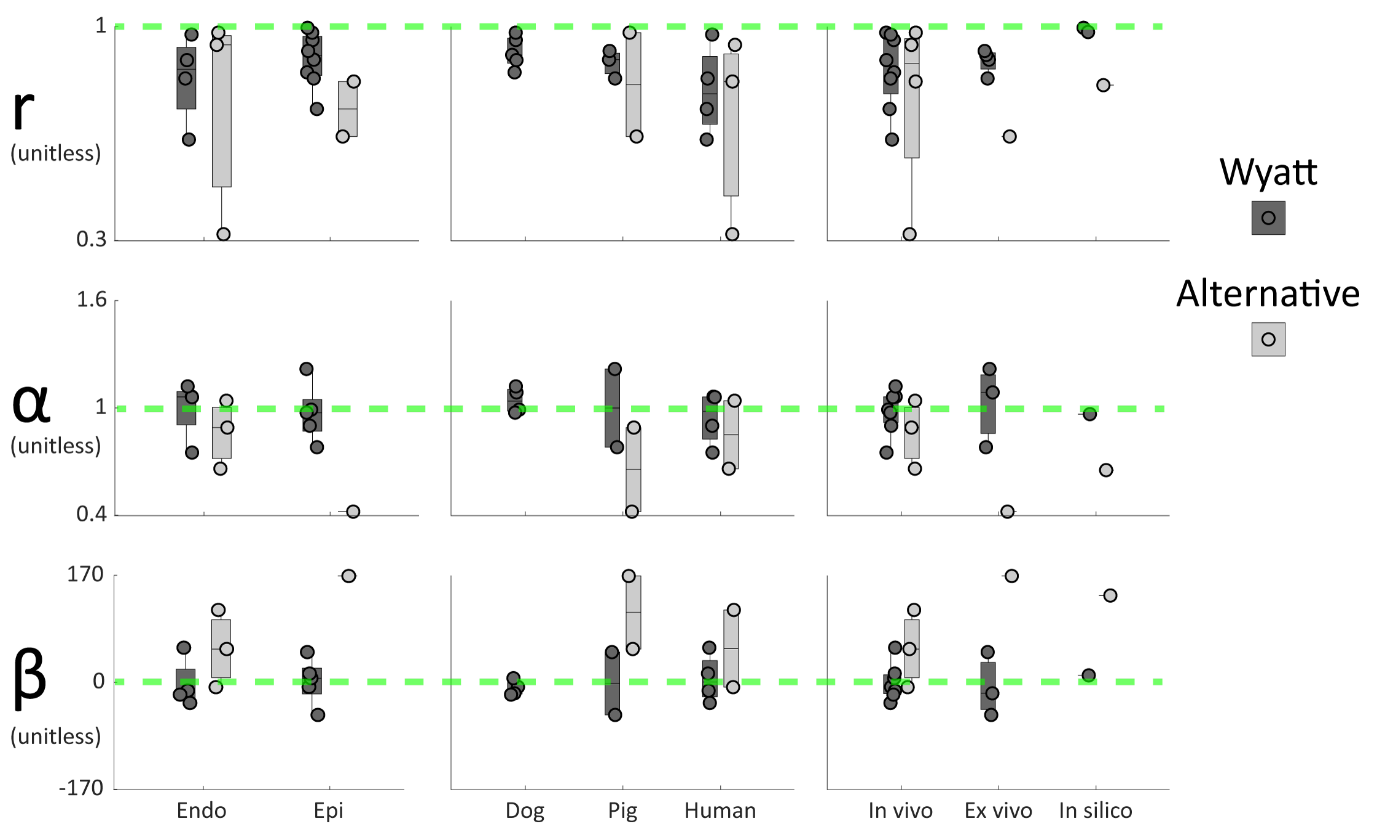 |
| --- |
| **Supplementary Figure 1.** Linear regression analyses for experimental results from previous studies in the literature ^4–8,14–16,19–21,38–40^. As in Figure 6, the accuracy of the Wyatt and alternative method were determined with respect to a local measure by addressing ARI= α ⋅<local measure>+ β, e.g. ARI= α ⋅MAPD_90_+β. Results were separated based on endocardial/epicardial approach (left column), species (middle column) and ex vivo/in silico/in vivo studies (right column). As visible, the Wyatt method more accurately reflects RT (defined by local measure) than the alternative method, even if analyses were limited to certain experimental setups, meaning that this is a globally true conclusion, no matter the conditions (species, site of measurement, or type of experiment). |

| 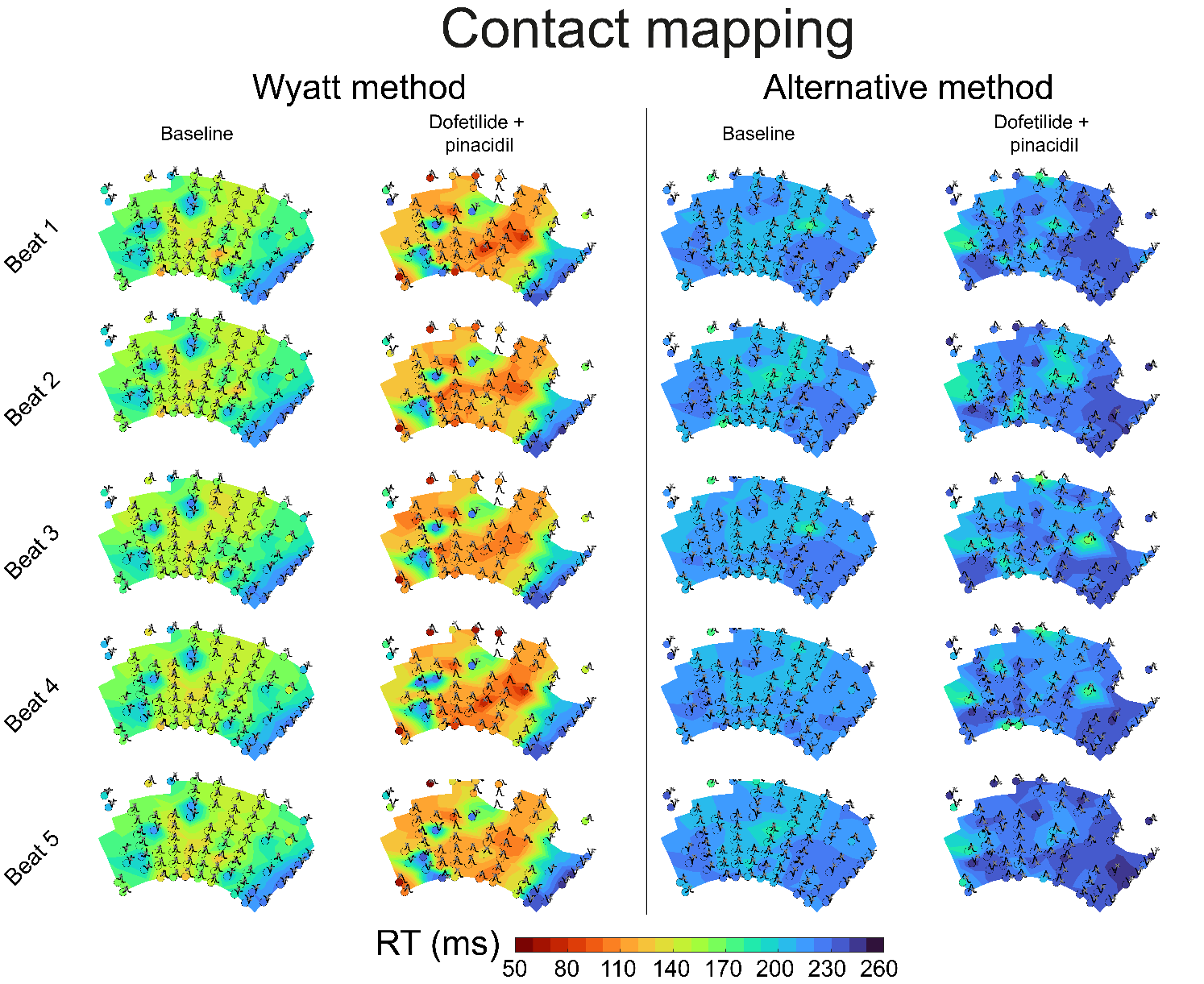 |
| --- |
| **Supplementary Figure 2.** Beat-to-beat consistency of RT determination through the Wyatt method and alternative method. Isochronal RT maps for five consecutive beats are shown for one of our experiments (experiment 13, see Figure 3). Each row denotes a beat, while each column denotes a drug setting. Isochronal RT maps are consistent, although RT determination may locally be sensitive to slight changes in maximum T-wave upslope. However, standard deviations of beat-to-beat RTs were not significantly different between the Wyatt and alternative method (p=0.84 for baseline, p=0.19 for the drug-infused situation). |
